# La prise en charge à domicile du paludisme chez l'enfant de 0 à 5 ans: Un problème réel de santé publique à Lubumbashi (RD Congo)

**DOI:** 10.11604/pamj.2014.18.214.4733

**Published:** 2014-07-15

**Authors:** Augustin Mulangu Mutombo, Gray A Wakamb Kanteng, Kristel Nzeba Tshibanda, Toni Kasole Lubala, Maguy Nsangaji Kabuya, Stanis Okitosho Wembonyama, Oscar Numbi Luboya

**Affiliations:** 1 Département de Pédiatrie, Université de Lubumbashi, Lubumbashi, République Démocratique du Congo

**Keywords:** Paludisme, prise en charge à domicile, Lubumbashi, malaria, Home-based management, Lubumbashi

## Aux éditeurs du Journal Panafricain de Médecine

Le paludisme est une cause majeure de mortalité et de morbidité chez l'enfant en zone d'endémie et constitue de nos jours un problème majeur de santé publique dans les pays en développement, notamment intertropicaux. Il est la plus grande endémie parasitaire avec 300 à 500 millions de personnes infectées à travers le monde et une mortalité annuelle estimée à entre 1,5 et 2,7 millions d′individus, touchant surtout les enfants [1]. En Afrique subsaharienne, le paludisme représente une des premières causes de morbidité et de mortalité chez l'enfant surtout chez ceux de moins de 5 ans [2]. A lui seul, le paludisme représente 10% de la charge totale de morbidité du continent africain [3].

Selon une enquête de l’école de santé publique de Kinshasa, en République Démocratique du Congo, le paludisme constitue la première cause de morbidité et de mortalité infanto-juvénile. Il est responsable de 67% des consultations externes et de 47,3% de décès d'enfants de moins de 5 ans [4]. En effet, un enfant congolais de moins de 5 ans connaît entre 6 à 10 épisodes de fièvre d'origine palustre par an [5].

Afin de lutter contre cette endémie, le programme national de lutte contre le paludisme en RDC(PNLP) a mis en place différentes stratégies pour le contrôle du paludisme tel que proposé par l'OMS [6]. Malgré la disponibilité des fonds et l'existence des moyens de lutte, le paludisme continue remplir les hôpitaux et a tuer les enfants de moins de 5 ans.

Dans le but d’étudier les attitudes et pratiques de la prise en charge à domicile du paludisme chez les enfants de moins de 5 ans, une étude d'observation descriptive et transversale a été effectuée dans l'aire de santé de Kalubwe situé dans la zone de santé de Lubumbashi au cours de la période allant du 4 Mai au 5 juin 2013. L’étude a ciblé les femmes de la zone de l'aire de santé de Kalubwe ayant les enfants de moins de 5 ans et qui étaient présentes lors de notre enquête. Au total 200 ménages ont été retenus et l'interview libre et structurée a été utilisée comme technique de collecte des données.

Les résultats ont révélé que la moyenne d’âge des mères interrogée était de 28±4, tandis que celle des enfants étaient de 34,1 mois, près de 90% des mères avaient plus d'un enfant et la majorité d'entre elles soit 83% étaient au moins diplômé d’état ce qui laisse entendre qu'elles avaient un niveau d'instruction pouvant leur permettre d'avoir jugement adéquat face à un problème sanitaire. Cependant, 42% d'entre elles ne connaissent aucune mesure de lutte contre le paludisme et seulement 48,5% de toutes les femmes enquêtées utilisent les moustiquaires. Au Togo, une étude menée sur la connaissance, attitude et pratique sur les méthodes de lutte contre le paludisme avait révélé que 65% des ménages avaient une connaissance sur les méthodes de lutte contre le paludisme, 51% avaient une bonne attitude et 39% avaient une bonne pratique de ses méthodes [7]. Et l’étude conclut qu'il existait une relation significative entre le niveau de connaissances et les pratiques, ce qui est différent de notre constat car le niveau d'instruction n’étaient pas significativement lié à la connaissance des méthodes de lutte contre le paludisme (p = 0,99) (Tableau 1).

**Tableau 1:** Répartition des moyens de prévention utilisés versus le niveau d'instruction

Moyen de prévention utilisé									
Niveau d'instruction		Moustiquaire	Insecticide en aérosol	Pommade révulsive	Assainissement du milieu	Serpentin insecticide	Aucun	TOTAL	p
	0 années d’études	3 (60%)	-	-	-	-	2 (40%)	5	0,9957
	< 6ans d’études	4 (50%)	-	-	3 (37,5%)	-	1 (12,5%)	8	
	≥ 6 ans d’études	46 (51,1%)	1 (1,1%)	-	7 (7,7%)	16 (17,7%)	20 (22,2%)	90	
	≥ 12 ans d’études	44 (52,4%)	6 (7,1%)	4 (4,8%)	6 (7,1%)	7(8,3%)	17 (20,2%)	84	

En interrogeant les mères sur la connaissance des symptômes du paludisme, 97,5% ont désigné la fièvre en première position, 34,5% ont donné les vomissements en deuxième position et 18% ont parlé des céphalées en troisième position.

Concernant la prise en charge médicamenteuse, la majorité utilise les antipaludéens 83,5% et c'est la quinine est la molécule la plus consommée dans 79% suivi des ACT dans 8% des cas et la chloroquine dans 5% des cas. Nos résultats sont de loin différents de ceux trouvés lors d'une étude CAP en Zone rurale Sénégalaise sur le paludisme qui permit d'observer que le traitement par la quinine occupait 60,2% des ménages suivi de l'artesunate-Amodiaquine qui représentaient 20,7% et 9,7% pour l'Artemether, le Fansidar avec 33,1% et 15,6% de Co-arinate et le Malaxin avait 38,2% dans la ville de Dakar [8]. En effet, cette situation de non-respect des standards et algorithmes du Ministère de Santé Publique de notre pays pourrait être responsable d'une pression médicamenteuse susceptible de créer les phénomènes de résistance pouvant lourdement affecter même la quinine et les combinaisons d'Amodiaquine-artemether. Il est à ce jour surprenant de retrouver en circulation la chloroquine alors que la politique nationale à retirer cette molécule du schéma de prise en charge du paludisme simple.

L'analyse des motivations de l'automédication chez nos enquêtées a montré que plusieurs mères soit 31,5% ont avouées le faire par manque des moyens et la cure de quinine étant moins de deux dollars américains, il est donc pour elles souhaitable de tenter une prise en charge à moindre cout avant de penser à consulter lors de la complication.

En effet, pour raisons de persistance des symptômes et parfois même aggravation du tableau clinique, 95 mères soit 47,5% étaient obligées de consulter à l'hôpital après un délais 3 à 6 jours ou le diagnostic de paludisme était retenu que dans 79,5% de cas. Ceci porte à croire que, le traitement antipaludique pris en ambulatoire avant la consultation n'a pas était efficace et cela pourrait probablement être expliqué soit par un sous dosage des médicaments par les parents ou encore par la prise des produits inadaptés. Nos résultats sur les délais de consultation se rapprochent de ceux de MABIALA qui lui a trouvé un délai de 1à 6 jours [9]. Signalons que, plus le temps du malade est long, plus le risque d’évoluer vers la sévérité devient élevé.

## Conclusion

Il ressort clairement que la perception et la prise en charge du paludisme à domicile chez les enfants de 0 à 5 ans sont délétères. En effet, la conception culturelle, les habitudes héritées et transmises de bouche à oreille jouent un rôle prépondérant, plutôt que le niveau d’éducation. Il est important que le programme national de lutte contre le paludisme procède par des campagnes de sensibilisation pour améliorer la perception du paludisme dans la population. Car les premiers gestes posés dès la maison, s'ils sont bien conduits, peuvent contribuer à réduire la mortalité.
